# Sample size and power determination when limited preliminary information is available

**DOI:** 10.1186/s12874-017-0329-1

**Published:** 2017-04-26

**Authors:** Christine E. McLaren, Wen-Pin Chen, Thomas D. O’Sullivan, Daniel L. Gillen, Min-Ying Su, Jeon H. Chen, Bruce J. Tromberg

**Affiliations:** 1 Department of Epidemiology, University of California, Irvine, 224 Irvine Hall, Irvine, CA USA; 2 Chao Family Comprehensive Cancer Center, University of California, Orange, CA USA; 3 Laser Microbeam and Medical Program, Beckman Laser Institute and Medical Clinic, University of California, Irvine, CA USA; 4 Department of Electrical Engineering, University of Notre Dame, Notre Dame, IN USA; 5 Department of Statistics, University of California, Irvine, CA USA; 6 Tu & Yuen Center for Functional Onco-Imaging, Department of Radiological Sciences, University of California Irvine, Irvine, CA USA

**Keywords:** Power and sample size calculation, Tamoxifen, Breast density, Magnetic resonance imaging, Diffuse optical spectroscopic imaging

## Abstract

**Background:**

We describe a novel strategy for power and sample size determination developed for studies utilizing investigational technologies with limited available preliminary data, specifically of imaging biomarkers. We evaluated diffuse optical spectroscopic imaging (DOSI), an experimental noninvasive imaging technique that may be capable of assessing changes in mammographic density. Because there is significant evidence that tamoxifen treatment is more effective at reducing breast cancer risk when accompanied by a reduction of breast density, we designed a study to assess the changes from baseline in DOSI imaging biomarkers that may reflect fluctuations in breast density in premenopausal women receiving tamoxifen.

**Method:**

While preliminary data demonstrate that DOSI is sensitive to mammographic density in women about to receive neoadjuvant chemotherapy for breast cancer, there is no information on DOSI in tamoxifen treatment. Since the relationship between magnetic resonance imaging (MRI) and DOSI has been established in previous studies, we developed a statistical simulation approach utilizing information from an investigation of MRI assessment of breast density in 16 women before and after treatment with tamoxifen to estimate the changes in DOSI biomarkers due to tamoxifen.

**Results:**

Three sets of 10,000 pairs of MRI breast density data with correlation coefficients of 0.5, 0.8 and 0.9 were simulated and generated and were used to simulate and generate a corresponding 5,000,000 pairs of DOSI values representing water, ctHHB, and lipid. Minimum sample sizes needed per group for specified clinically-relevant effect sizes were obtained.

**Conclusion:**

The simulation techniques we describe can be applied in studies of other experimental technologies to obtain the important preliminary data to inform the power and sample size calculations.

**Electronic supplementary material:**

The online version of this article (doi:10.1186/s12874-017-0329-1) contains supplementary material, which is available to authorized users.

## Background

Mammographic density, an assessment of the fibroglandular content of the breast using mammography, is an important indicator of breast cancer risk and hormonal-based treatment response. Women with high mammographic density have from 4- to 6-fold increased breast cancer risk compared to women with lower density [1]. There is also significant evidence that the selective estrogen receptor modulator tamoxifen is more effective at reducing breast cancer risk when accompanied by a reduction of mammographic density [2–5]. However, due to the limitations of using mammography for frequent, quantitative breast density assessment [6], we sought to test whether other imaging modalities can better quantify breast density. Magnetic resonance imaging (MRI) is a safe and quantitative technique for measuring breast density and volume, but its high cost precludes frequent, widespread use in risk assessment or therapeutic monitoring. As an alternative to MRI and mammography, diffuse optical spectroscopic imaging (DOSI) is a non-invasive, relatively low-cost experimental imaging technique that provides quantitative metrics to measure and track changes in breast tissue composition and metabolism [7]. In previous research, we measured the breast density and composition of normal breast from 12 volunteers’ contralateral normal side of breast cancer before and during neoadjuvant chemotherapy (NAC) treatment with MRI and DOSI. The strong significant linear relationship between the MRI-based breast density and these DOSI measures including water, deoxygenated hemoglobin (ctHHb) and lipid volume was reported [8]. This finding suggests that these DOSI imaging biomarkers are useful for monitoring changes in breast tissue composition and density. However further quanification of the association between within-subject changes in DOSI measures and changes in baseline is needed before DOSI measures can be considered as an alternative biomarker in treatment settings.

**Fig. 1:**
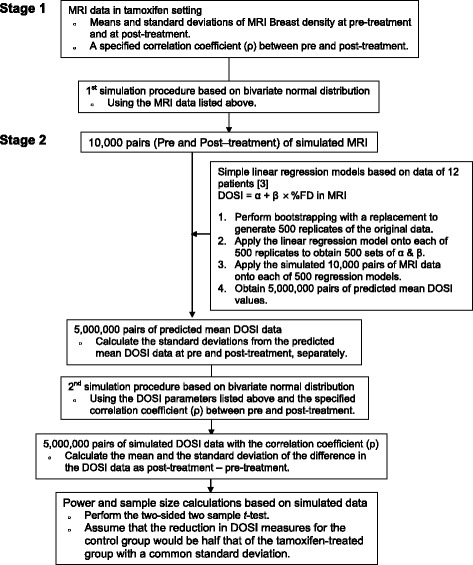
Study Schema: A flow chart display of the simulation procedures

In order to design a study to compare the reduction from baseline in DOSI measures that may reflect changes in breast density in premenopausal women receiving tamoxifen and a control group, preliminary information of DOSI measures in a tamoxifen treatment setting are needed to inform power and sample size calculations. While direct information on DOSI in this case is not available, measures of changes in MRI-based breast density have previously been reported. Chen and colleagues reported breast density measurements assessed by three-dimensional (3D) MRI before and after tamoxifen treatment among sixteen patients [9]. Utilizing these data along with cross-sectional estimates of the relationship between DOSI imaging biomarkers and MRI density could help to provide more informative uncertainty estimates for the design of studies to assess within-subject DOSI changes. Specifically, we developed a statistical simulation approach utilizing information from MRI assessment of breast density before and after treatment with tamoxifen [9] and a separate study of DOSI images obtained from volunteers about to begin neoadjuvant chemotherapy [8]. We applied a two-stage strategy that enhances the validity of the power and sample size calculations in an observational study where there is a lack of direct preliminary data [10]. A schematic of our two-stage procedure involving the simulation approach is shown in Fig. 1. The simulation alogrithm was programmed utlizing SAS^©^ software, Version 9.4 [11], and is provided in the Additional files.

## Methods

### DOSI measures

**Fig. 2:**
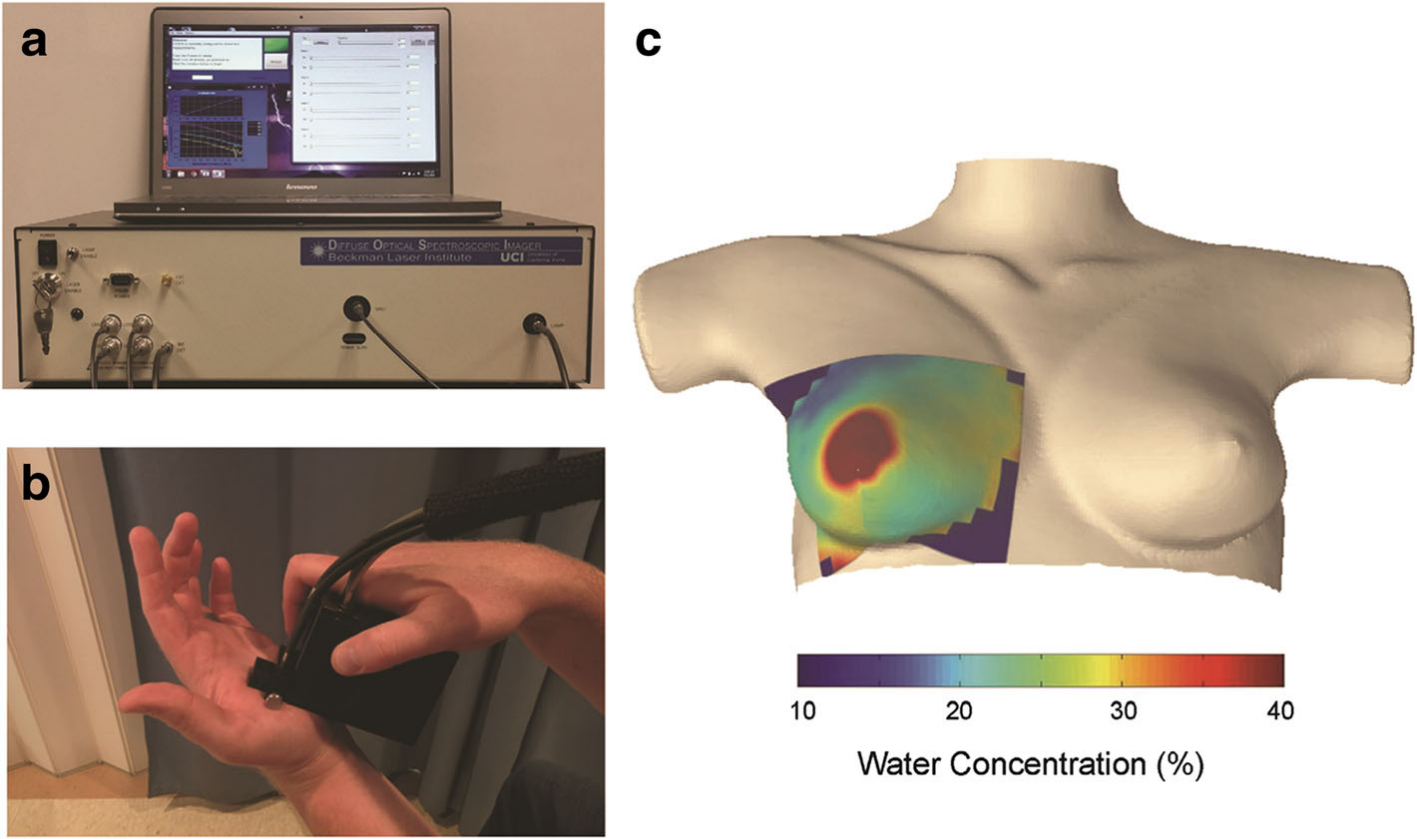
Diffuse Optical Spectroscopic Imaging (DOSI): **a** Portable, bedside DOSI instrument, **b** Handheld DOSI probe that is scanned across the breast to collect data, and **c** DOSI image of breast water concentration

A laser breast scanner has been developed based on DOSI technology (Fig. 2) [12]. Measurements were acquired before administration of chemotherapy and frequently during therapy through a handheld probe placed on the tissue surface. Acquisition times for single measurements are approximately five seconds. Measurements are representative of optical properties in a total tissue volume of approximately 100cm^3^. Subjects were measured at the Beckman Laser Institute and Medical Clinic, University of California, Irvine, CA. The contralateral normal breast of twelve patients with locally advanced breast cancer receiving neoadjuvant chemotherapy was measured with DOSI and MRI. The relevant DOSI measures for this study included percent water, percent ctHHb and percent lipid.

### Modeling the correspondence between DOSI biomarkers and MRI percent density

Simple linear regression models were formed with an individual DOSI biomarkers at baseline as the response variable and the corresponding MRI breast density at baseline as the explanatory variable. Let *Y* be a given DOSI response variable and let *x* be the corresponding MRI percent density. Then the regression model can be written as follows:

1 $$ {Y}_i=\alpha +\beta {x}_i+{\varepsilon}_i,{\varepsilon}_i\sim N\left(0,{\sigma}^2\right), i=1,\dots, 12. $$

**Fig. 3:**
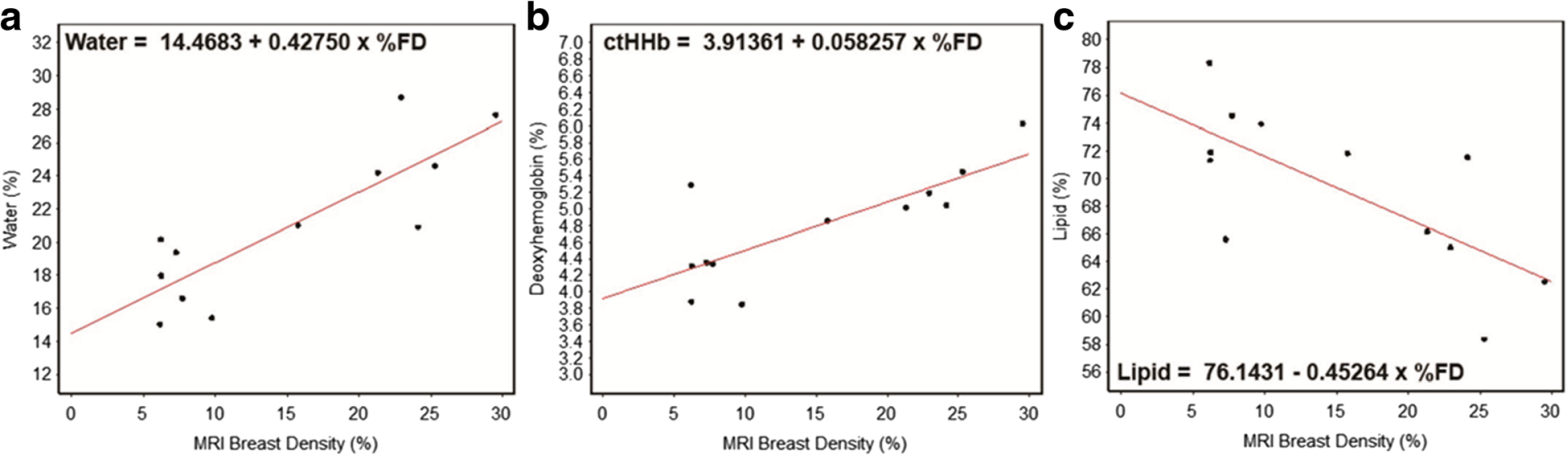
Linear regression models: **a** water versus MRI breast density, **b** deoxyhemoglobin (ctHHb) versus MRI breast density and **c** lipid versus MRI breast density

For each model, ordinary least squares was used to estimate the linear correlation (*ρ*) between *Y* and *x* and the *p*-value from the hypothesis test of Ho: *ρ* = 0 vs HA: *ρ* ≠ 0 were recorded. At a significance level of 0.05, there was a statistically significant linear correlation between MRI percent breast density prior to therapy and each of the three DOSI measures including percent water (estimated linear correlation *r* = 0.843, *p* < 0.001), μM of ctHHb (*r* = 0.785, *p* = 0.003) and lipid (*r* = −0.707, *p* < 0.001) (Table 1, Fig. 3).

**Table 1:** Correlation coefficients and *p*-values for DOSI measures versus MRI breast density among twelve patients

	Baseline (*N* = 12)	
	ρ	*p*-value
Water	0.843	<0.001
ctHHb	0.785	0.003
Lipid	−0.707	0.010

### Bootstrapping the DOSI measures

The simple linear regression models from Eq. (1) were based on the data from 12 subjects. In order to quantify variability in the estimates of the slope parameter in the model (*β*) while accounting for potential departures from the assumption of the classical normal linear regression model, the bootstrap was used [13, 14]. Specifically, the observations obtained on the 12 subjects were treated as a pseudo-population and sampling with replacement was utilized to obtain 500 replicate sets of data from the pseudo-population. The process was repeated for each DOSI biomarker and based upon the 500 pseudo-samples, an analogous regression model to that described in Eq. (1) was fit and the sampling distribution of the estimated slope and linear correlation coefficient were estimated.

### Simulated MRI breast density data in a tamoxifen treatment setting

Based on the study of Chen and colleagues [9], 16 women were enrolled and received 3D MRI assessment of breast density before and after tamoxifen treatment. The mean pre- and post-treatment percent density (standard deviation, SD) was 22.1% (2.6%) and 16.3% (3.3%), respectively. The estimated correlation coefficient between pre- and post-treatment was 0.9. Let *X*_*pre*_ represents the MRI percent breast density before the treatment and *Y*_*post*_ represents the MRI percent breast density after the treatment. Let *μ*_*x*_ and *μ*_*y*_ be the mean percent breast densities with corresponding standard deviations *σ*_*x*_ and *σ*_*y*_ measured pre- and post-treatment, respectively. The within-subject correlation between *X*_*pre*_ and *Y*_*post*_ is represented by *ρ*_MRI_. A bivariate normal probability density function for *X*_*pre*_ and *Y*_*post*_ is given in Eq. (2) and was assumed to simulate 10,000 pairs of MRI percent breast density data with a specified correlation between pre- and post-treatment values. The simulation procedure was repeated for each specified correlation coefficient of 0.5, 0.8 and 0.9, separately in order to reflect the strong linear association observed in Chen et al. as well as two less optimistic cases.

2 $$ f\left( x, y\right)=\frac{1}{2\pi {\sigma}_x{\sigma}_y\sqrt{1-{\rho}^2}} \exp \left[-\frac{1}{2\left(1-{\rho}^2\right)}\left[{\left(\frac{x-{\mu}_x}{\sigma_x}\right)}^2+{\left(\frac{y-{\mu}_y}{\sigma_y}\right)}^2-2\rho \left(\frac{x-{\mu}_x}{\sigma_x}\right)\left(\frac{y-{\mu}_y}{\sigma_y}\right)\right]\right] $$

### Simulation of within-subject changes in DOSI values

To account for uncertainty in both the association between pre- and post-MRI density measures and the cross-sectional relationship between a given DOSI outcome and MRI-based density, the following algorithm was implemented.

1. 500 bootstrap estimates of the cross-sectional linear relationship between the DOSI measurement and MRI density were obtained, as described in the previous section entitled “Modeling the correspondence between DOSI biomarkers and MRI percent density”.
2. For each assumed correlation (0.5, 0.8 and 0.9), 10,000 simulated pre- and post-MRI density pairs were simulated from the bivariate model described in the previous section entitled Bootstrapping the DOSI measures.
3. For each of the bootstrap estimates obtained in Step 1, the 10,000 pre- and post-simulated MRI density values from Step 2 were used to generate a corresponding simulated mean DOSI value via the simple linear regression model given in Eq (1).

In the above algorithm we assume that the observed linear relationship between pre-treatment MRI and DOSI values would also hold for post-treatment values, and the correlation coefficient in MRI and DOSI between pre and post-therapy was consistent. Using this approach, we obtained predicted mean DOSI values at baseline and after treatment and the standard deviations of predicted DOSI values for each simulated case. To take into account the correlation between estimated pre- and post-treatment DOSI values, the predicted mean and SD for percent water, μM ctHHb and percent lipid measured at baseline and after treatment were then used in a second simulation of bivariate normal distributions with specified correlation coefficients of 0.5, 0.8, and 0.9. This resulted in 5,000,000 corresponding pairs of pre- and post-therapy values generated for each of the three DOSI measures (percent water, μM ctHHb and percent lipid) at each of specified correlation coefficients. The SAS programming code for our application of a two-stage strategy is available (see Additional file 1: Supplemental SAS program).

### Preliminary data for power and sample size calculation

Pre- and post-treatment differences between values for each DOSI biomarker were calculated and used to inform power and sample size calculations for a two-sample *t*-test of the mean reduction from baseline in the tamoxifen-treated vs. control groups with a specified power and significance level. Since a study of tamoxifen-induced reduction of mammographic density reported that the mean reduction in density among placebo control subjects was approximately half the mean density reduction in tamoxifen-treated subjects (3.5% vs 7.9%), we assumed that the clinically-relevant reduction in DOSI measures in the control group would be half that of the tamoxifen-treated group [2, 8, 15]. The program nQuery v7.0 was utilized for power and sample size determinations [16].

## Results

**Fig. 4:**
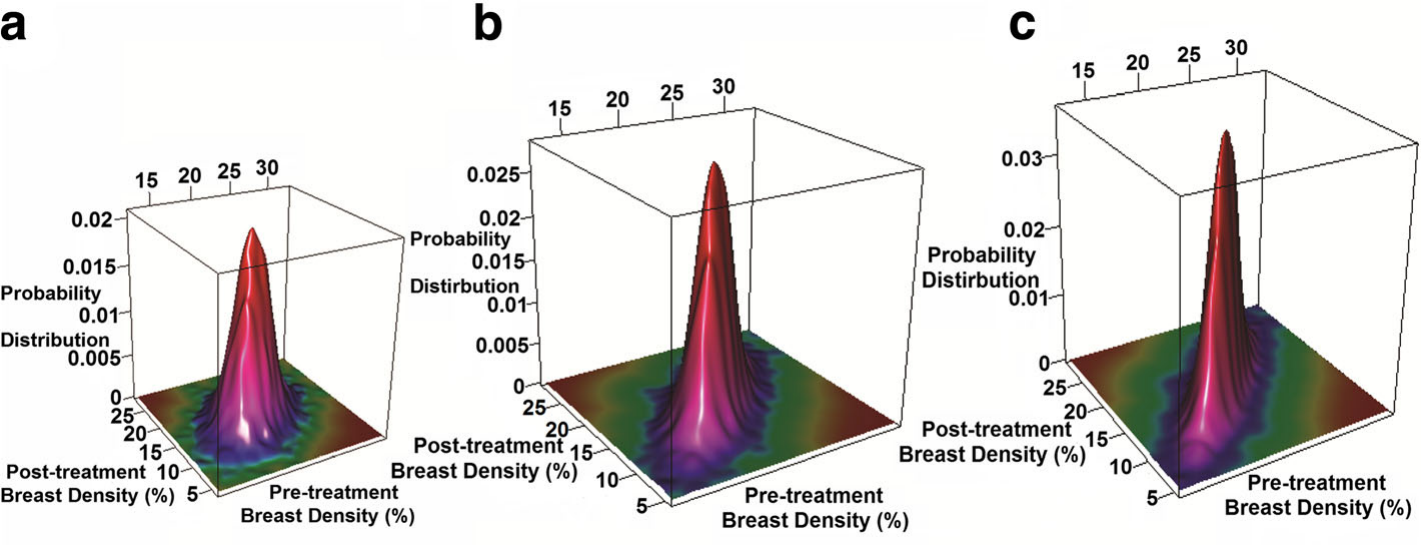
Probability distribution plots of 10,000 simulated MRI breast density data representing pre- and post-tamoxifen treatment assuming the following: **a** correlation coefficient of 0.5, **b** a correlation coefficient of 0.8, and **c** a correlation coefficient of 0.9

Results of the simulation procedures are displayed in Fig. 4. Three sets of 10,000 pairs of MRI breast density data with correlation coefficient of 0.5, 0.8 and 0.9 were simulated and generated, respectively as described in the study schema (Fig. 1). These were used to simulate and generate a corresponding 5,000,000 pairs of DOSI values representing water, ctHHb, and lipid (Table 2). Based on the simulated DOSI data, the observed means and SDs of the changes in the treated group were listed in Table 2. The estimated sample size of participants per group needed to detect a mean reduction in the control group of half that of the treated group for a specified power and a significance level are listed in Table 3. For example, under the assumptions of the correlation coefficient between pre- and post-treatment with a value of 0.50 and the common standard deviation for the treated and control groups, the observed mean changes and SD of the changes in the treated group were -2.5% (2.12%), −0.3μM (0.29μM) and 2.6% (2.63%) for water, ctHHB, and lipid respectively, compared to −1.3% (2.12%), −0.2μM (0.29μM), and 1.3% (2.63%) in the control group. With 80% power and a significance level of 0.05, the required sample sizes per group are 47 subjects for water, 48 for ctHHb, and 65 for lipid (Table 3). Additional file 2: Tables S1, S2, and S3 show details of the minimum sample size needed per group for specified clinically-relevant sample sizes, assuming significance levels of 0.05 and 0.01, power of 80 and 90%, and correlation coefficients between pre- and post-treatment values of water, ctHHB, and lipid of 0.50, 0.80, and 0.90.

**Table 2:** Simulated preliminary data for power and sample size calculations

Variable		Estimated correlation coefficient					
		0.50		0.80		0.90	
	N	Mean	STD^a^	Mean	STD^a^	Mean	STD^a^
The simulated MRI breast density at baseline data	10,000	22.1	2.59	22.1	2.59	22.1	2.59
The simulated MRI breast density at follow-up data	10,000	16.3	3.33	16.3	3.32	16.3	3.32
pre_ctH2O: Predicted mean and STD for 2nd simulation	5,000,000	23.8	1.53	23.8	1.53	23.8	1.53
post_ctH2O: Predicted mean and STD for 2nd simulation	5,000,000	21.3	1.64	21.3	1.64	21.4	1.63
Random selected baseline water data after applying the regression model	5,000,000	23.9	2.16	23.9	2.16	23.9	2.16
Random selected follow-up water data after applying the regression model	5,000,000	21.3	2.32	21.3	2.32	21.4	2.31
*Water: Follow-up data – Baseline data* ^b^	*5,000,000*	*-2.5*	*2.12*	*−2.5*	*1.42*	*−2.5*	*1.09*
pre_ctHHb: Predicted mean and STD for 2nd simulation	5,000,000	5.2	0.19	5.2	0.19	5.2	0.19
post_ctHHb: Predicted mean and STD for 2nd simulation	5,000,000	4.8	0.23	4.8	0.23	4.9	0.23
Random selected baseline ctHHb data after applying the regression model	5,000,000	5.2	0.26	5.2	0.26	5.2	0.26
Random selected follow-up ctHHb data after applying the regression model	5,000,000	4.8	0.32	4.9	0.32	4.9	0.32
*ctHHb: Follow-up data – Baseline data* ^b^	*5,000,000*	*−0.3*	*0.29*	*−0.3*	*0.20*	*−0.3*	*0.16*
pre_Lipid: Predicted mean and STD for 2nd simulation	5,000,000	66.3	2.06	66.3	2.06	66.3	2.06
post_Lipid: Predicted mean and STD for 2nd simulation	5,000,000	68.9	2.01	68.9	2.00	68.9	2.00
Random selected baseline Lipid data after applying the regression model	5,000,000	66.3	2.92	66.3	2.92	66.3	2.92
Random selected follow-up Lipid data after applying the regression model	5,000,000	68.9	2.84	68.9	2.84	68.9	2.83
*Lipid: Follow-up data – Baseline data* ^b^	*5,000,000*	*2.6*	*2.63*	*2.6*	*1.82*	*2.6*	*1.44*

^a^ *STD* standard deviation﻿

^b^The italic font indicates that the values were used in the sample size deteremination procedure

**Table 3:** Estimated sample size needed per group in various scenarios

DOSI Measure	Power	Significance level	Estimated correlation coefficient^a^					
			0.50		0.80		0.90	
			Common STD^b^ (σ)	2 × Common Variance	Common STD^b^ (σ)	2 × Common Variance	Common STD^b^ (σ)	2 × Common Variance
Water			σ = 2.121	σ = 2.999	σ = 1.420	σ = 2.008	σ = 1.088	σ = 1.539
	80	0.01	69	136	32	62	20	38
		0.05	47	92	22	42	13	25
	90	0.01	88	173	41	79	25	47
		0.05	62	122	29	56	17	33
Deoxyhemoglobin (ctHHb)			σ = 0.287	σ = 0.406	σ = 0.198	σ = 0.279	σ = 0.158	σ = 0.224
	80	0.01	72	142	35	68	23	45
		0.05	48	95	24	46	16	30
	90	0.01	90	180	45	86	29	56
		0.05	64	127	31	61	21	40
Lipid			σ = 2.633	σ = 3.724	σ = 1.816	σ = 2.568	σ = 1.443	σ = 2.041
	80	0.01	96	191	47	92	31	59
		0.05	65	128	32	62	21	40
	90	0.01	122	242	59	117	38	75
		0.05	86	171	42	80	27	53

^a^The correlation coefficient was estimated between pre- and post-treatment in simulated MRI data and in simulated DOSI measure

^b^The standard deviation is denoted as STD

## Discussion

When attempting to quantify the statistical operating characteristics of a proposed study design there is often little relevant preliminary data available to inform power and sample size determination. Because of this a common approach is to use indirect estimates of variability and effect size (at best) or assumed estimates in the absence of empirical data (at worst). As with the DOSI trial considered in this manuscript there may exist parameter estimates for established response variables that have been shown to be highly correlated with the novel outcome of interest being considered in the actual study. In this case, it is tempting to treat association estimated between the novel and established variables as fixed, but this approach fails to incorporate uncertainty in these estimates and hence may yield overly optimistic estimates of the planned study’s design operating characteristics.

In the example we have discussed, indirect measures of the distributional parameters for DOSI do exist and could be utilized to provide more valid estimates of sample size and power for a prospectively designed study. Specifically, we were able to utilize information on the cross-sectional association between DOSI biomarkers and MRI-based density outcomes together with separate information on the within-subject change in MRI-based outcomes. In order to account for uncertainty in the parameter estimates stemming from both sources of information a two-stage simulation approach was employed. As demonstrated, the simulation techniques we described can be applied to obtain the important preliminary data to inform the power and sample size calculations in such cases. Given the importance of realistic estimates of study design operating characteristics we view the approach provided here as a far superior method when compared to the usual simple assumptions that are often employed by study designers.

Multiple authors have considered power and sample size estimation. A fairly comprehensive approach to sample size estimation for standard 1- and 2-sample problems can be found in Van Belle et al. [17]. In addition, Lenth [18] provides practical guidance for determining the parameters to be used in sample size estimation. In the context of linear regression, Hsieh et al. [19] provides closed form solutions when parameter values are assumed under fairly simple settings with limited numbers of adjustment covariates. In more complex scenarios, simulation is generally required in order to capture the correlation structure across adjustment covariates. Burton et al. [20] provide comprehensive guidelines for designing and implementing simulation studies, with applications to sample size estimation for logistic and survival models. In the context of simulated sample size determination for specific applications, Desmond and Glover [21] consider the use of simulation for sample size determination in the context of fMRI imaging studies. In their work, parameter values used in the simulation were derived from observed pilot data. Haneuse et al. [10] have further a two-stage approach that utilizes simulation of statistical operating characteristics both at the design phase of a study and later seeks to incorporate updated correlation and variance estimates after preliminary data collection has been obtained. They did not consider the use of indirect association estimates as we have provided here, though the techniques presented in [10] could also be of use in the DOSI study after initial data collection has been obtained in order to update and further inform the statistical operating characteristics of the study. This remains an area of future work for this project.

## Conclusion

Many different breast imaging modalities, including mammography, MRI, optical imaging, ultrasound, computed tomography (CT), and nuclear medicine, can be used to measure breast density, as described in a recent review paper [22]. Although the underlying mechanisms to identify dense tissue in a breast were different by using different imaging methods; yet in general, due to the strong contrast between dense and fatty tissues, and the quantitative density measures done by using different imaging modalities were highly correlated. This offers a great opportunity to obtain a good estimate of effect size when designing a new study by using the density measured by more-established methods, such as mammography and MRI. In this work, the reduction of density in subject receiving tamoxifen treatment was measured by MRI, and the effect size along with the measurement variation of DOSI could be used to do a realistic power analysis. This strategy can be potentially applied to many other imaging studies done by using novel imaging methods that do not have sufficient preliminary results, based on the high correlation with results obtained by using established imaging modalities. Our two-stage approach provides a feasible framework.

## Additional files

Additional file 1: SAS Program Code. (DOCX 50 kb)

Additional file 2: Table S1. Sample sizes needed per group for a two-sided two group *t*-test of equal means assuming a significance level of 0.05 or 0.01, power of 80% or 90%, correlation coefficient between pre and post-treatment values of 0.50 for each DOSI measure, and equal sample sizes per group*. **Table S2.** Sample sizes needed per group for a two-sided two group *t*-test of equal means assuming a significance level of 0.05 or 0.01, power of 80% or 90%, correlation coefficient between pre and post-treatment values of 0.80 for each DOSI measure, and equal sample sizes per group. **Table S3.** Sample sizes needed per group for a two-sided two group *t*-test of equal means assuming a significance level of 0.05 or 0.01, power of 80% or 90%, correlation coefficient between pre and post-treatment values of 0.90 for each DOSI measure, and equal sample sizes per group. (DOC 109 kb)

## Abbreviations

3D: Three-dimensional
CT: Computed tomography
ctHHb: Deoxygenated hemoglobin
DOSI: Diffuse optical spectroscopic imaging
MRI: Magnetic resonance imaging
NAC: Neoadjuvant chemotherapy
SD: Standard deviation
