# Supplementary material for: Sample size and power determination when limited preliminary information is available
Source: BMC Med Res Methodol. 2017 Apr 26;17:75. doi: 10.1186/s12874-017-0329-1 (PMC5406943; doi:10.1186/s12874-017-0329-1)
Supplement: Supplementary file 1 — SAS Program Code. (DOCX 50 kb) [file 12874_2017_329_MOESM1_ESM.docx]

**Additional file 1**

**SAS Program Code**

libname h "C:\Temp";

** Simulated 10000 MRI data;

**%macro** sim(outdt=,rho=);

data &outdt;

keep BD0 BD1;

BL=**22.1**; FU=**16.3**; BL_std=**2.6**; FU_std=**3.3**; rho=&rho;

BL_var=BL_std****2**; FU_var=FU_std****2**;

c=sqrt(**1**-rho****2**);

do i = **1** to **10000**;

BD0 = rannor(**123**);

BD1 = rho*BD0+c*rannor(**123**);

BD0 = BL + BL_std*BD0;

BD1 = FU + FU_std*BD1;

output;

end;

run;

**%mend**;

** Bootstrapping real data (N=12) for 500 replicate sets and perform 500 regression models;

**%macro** dt(y=,time=,simdt=,rho=,testdt=);

ods output ParameterEstimates=estout;

proc reg data = h.boot500sample outest=out_&y;

by replicate;

hat_&y: model &y=__FD;

run;

quit;

dm log 'clear';

dm output 'clear';

data estout1(keep=intercept intercept_std k replicate); set estout;

if Variable='Intercept' then do ; intercept=Estimate;

intercept_std=StdErr; k=**1**; output; end;

data estout2(keep=Coeff Coeff_std k replicate); set estout;

if Variable='__FD' then do; Coeff=Estimate; Coeff_std=StdErr; k=**1**;

output; end;

run;

data coeff; merge estout1 estout2; by replicate; run;

** Use Simulated 10000 MRI data to applied each of 500 regression models;

** Total 10000 x 500 pairs of DOSI data;

%do i=**1** %to **500**;

data temp1; set &simdt; replicate=&i;

data temp1out; merge temp1(in=in1) coeff(in=in2); by replicate; if in1 and in2 then output; run;

data temp1out1; set temp1out;

pre_&y=intercept+Coeff*BD0;

post_&y=intercept+Coeff*BD1;

run;

proc append data=temp1out1 base=&testdt force; run;

dm log 'clear';

dm output 'clear';

%end;

proc means data=&testdt n std; var pre_&y post_&y;

output out=margin_std

std=pre_std post_std;

run;

data margin_std1(keep= pre_std post_std g); set margin_std; g=**1**; run;

data &testdt; set &testdt; g=**1**; run;

data Temp2; merge &testdt margin_std1; by g;

c=sqrt(**1**-&rho****2**);

Do Until(prenew_&y>=**0** and postnew_&y>=**0**);

p0 = rannor(**123**);

p1 = &rho*p0+c*rannor(**123**);

if "&y"="Lipid" then do;

prenew_&y = pre_&y - (pre_std)*p0;

postnew_&y = post_&y - (post_std)*p1;

end;

if "&y"~="Lipid" then do;

prenew_&y = pre_&y + (pre_std)*p0;

postnew_&y = post_&y + (post_std)*p1;

end;

end;

run;

quit;

data h.DT_&y&time; set Temp2; d_&y=postnew_&y - prenew_&y; run; quit;

**%mend**;
