# Supplementary material for: Sample size and power determination when limited preliminary information is available
Source: BMC Med Res Methodol. 2017 Apr 26;17:75. doi: 10.1186/s12874-017-0329-1 (PMC5406943; doi:10.1186/s12874-017-0329-1)
Supplement: Supplementary file 2 — Sample sizes needed per group for a two-sided two group t-test of equal means assuming a significance level of 0.05 or 0.01, power of 80% or 90%, correlation coefficient between pre and post-treatment values of 0.50 for each DOSI measure, and equal sample sizes per group*. Table S2. Sample sizes needed per group for a two-sided two group t-test of equal means assuming a significance level of 0.05 or 0.01, power of 80% or 90%, correlation coefficient between pre and post-treatment values of 0.80 for each DOSI measure, and equal sample sizes per group. Table S3. Sample sizes needed per group for a two-sided two group t-test of equal means assuming a significance level of 0.05 or 0.01, power of 80% or 90%, correlation coefficient between pre and post-treatment values of 0.90 for each DOSI measure, and equal sample sizes per group. (DOC 109 kb) [file 12874_2017_329_MOESM2_ESM.doc]

**Additional file 2**

**Table S1**. Sample sizes needed per group for a two-sided two group *t*-test of equal means assuming a significance level of 0.05 or 0.01, power of 80% or 90%, correlation coefficient between pre and post-treatment values of 0.50 for each DOSI measure, and equal sample sizes per group.*

| **Scenario** | ctH2O | | ctHHb | | Lipid | |
| --- | --- | --- | --- | --- | --- | --- |
| Treatment Group mean, μ_1_ | -2.504 | -2.504 | -0.332 | -0.332 | 2.623 | 2.623 |
| Control Group mean, μ_2_ = μ_1_ **0.5** | -1.252 | -1.252 | -0.166 | -0.166 | 1.312 | 1.312 |
| Difference in means, μ_1_ - μ_2_ | -1.252 | -1.252 | -0.166 | -0.166 | 1.311 | 1.311 |
| Common standard deviation, σ | 2.121 | 2.999 | 0.287 | 0.406 | 2.633 | 3.724 |
| Effect size, d = | μ_1_ - μ_2_| / σ | 0.590 | 0.417 | 0.578 | 0.409 | 0.498 | 0.352 |
| Test significance level, α | 0.01 | 0.01 | 0.01 | 0.01 | 0.01 | 0.01 |
| Power ( % ) | 80 | 80 | 80 | 80 | 80 | 80 |
| **n per group** | **69** | **136** | **72** | **142** | **96** | **191** |
| Test significance level, α | 0.05 | 0.05 | 0.05 | 0.05 | 0.05 | 0.05 |
| Power ( % ) | 80 | 80 | 80 | 80 | 80 | 80 |
| **n per group** | **47** | **92** | **48** | **95** | **65** | **128** |
| Test significance level, α | 0.01 | 0.01 | 0.01 | 0.01 | 0.01 | 0.01 |
| Power ( % ) | 90 | 90 | 90 | 90 | 90 | 90 |
| **n per group** | **88** | **173** | **90** | **180** | **122** | **242** |
| Test significance level, α | 0.05 | 0.05 | 0.05 | 0.05 | 0.05 | 0.05 |
| Power ( % ) | 90 | 90 | 90 | 90 | 90 | 90 |
| **n per group** | **62** | **122** | **64** | **127** | **86** | **171** |

*Calculated values have been rounded to three decimal places.

**Assume the following: (1) a common standard deviation σ_1_ and variance σ_1_^2^ based on simulated data or (2) a common variance σ_2_^2^ that is twice that of σ_1_^2^. Example: let σ_1_=2.121, then σ_2_ ^2^= 2σ_1_^2^ = 8.997, and σ_2 =_ 2.999.

**Table S2**. Sample sizes needed per group for a two-sided two group *t*-test of equal means assuming a significance level of 0.05 or 0.01, power of 80% or 90%, correlation coefficient between pre and post-treatment values of 0.80 for each DOSI measure, and equal sample sizes per group.

| **Scenario** | ctH2O | | ctHHb | | Lipid | |
| --- | --- | --- | --- | --- | --- | --- |
| Treatment Group mean, μ_1_ | -2.501 | -2.501 | -0.332 | -0.332 | 2.621 | 2.621 |
| Control Group mean, μ_2_ = μ_1_ 0.5 | -1.251 | -1.251 | -0.166 | -0.166 | 1.311 | 1.311 |
| Difference in means, μ_1_ - μ_2_ | -1.250 | -1.250 | -0.166 | -0.166 | 1.310 | 1.310 |
| Common standard deviation, σ | 1.420 | 2.008 | 0.198 | 0.279 | 1.816 | 2.568 |
| Effect size, d = | μ_1_ - μ_2_| / σ | 0.880 | 0.623 | 0.838 | 0.595 | 0.721 | 0.510 |
| Test significance level, α | 0.01 | 0.01 | 0.01 | 0.01 | 0.01 | 0.01 |
| Power ( % ) | 80 | 80 | 80 | 80 | 80 | 80 |
| **n per group** | **32** | **62** | **35** | **68** | **47** | **92** |
| Test significance level, α | 0.05 | 0.05 | 0.05 | 0.05 | 0.05 | 0.05 |
| Power ( % ) | 80 | 80 | 80 | 80 | 80 | 80 |
| **n per group** | **22** | **42** | **24** | **46** | **32** | **62** |
| Test significance level, α | 0.01 | 0.01 | 0.01 | 0.01 | 0.01 | 0.01 |
| Power ( % ) | 90 | 90 | 90 | 90 | 90 | 90 |
| **n per group** | **41** | **79** | **45** | **86** | **59** | **117** |
| Test significance level, α | 0.05 | 0.05 | 0.05 | 0.05 | 0.05 | 0.05 |
| Power ( % ) | 90 | 90 | 90 | 90 | 90 | 90 |
| **n per group** | **29** | **56** | **31** | **61** | **42** | **80** |

*Calculated values have been rounded to three decimal places.

**Assume the following: (1) a common standard deviation σ_1_ and variance σ_1_^2^ based on simulated data or (2) a common variance σ_2_^2^ that is twice that of σ_1_^2^. Example: let σ_1_=1.420, then σ_2_ ^2^= 2σ_1_^2^ = 4.033, and σ_2 =_ 2.008.

**Table S3**. Sample sizes needed per group for a two-sided two group *t*-test of equal means assuming a significance level of 0.05 or 0.01, power of 80% or 90%, correlation coefficient between pre and post-treatment values of 0.90 for each DOSI measure, and equal sample sizes per group.

| **Scenario** | ctH2O | | ctHHb | | Lipid | |
| --- | --- | --- | --- | --- | --- | --- |
| Treatment Group mean, μ_1_ | -2.499 | -2.499 | -0.332 | -0.332 | 2.618 | 2.618 |
| Control Group mean, μ_2_ = μ_1_ 0.5 | -1.250 | -1.250 | -0.166 | -0.166 | 1.309 | 1.309 |
| Difference in means, μ_1_ - μ_2_ | -1.249 | -1.249 | -0.166 | -0.166 | 1.309 | 1.309 |
| Common standard deviation, σ | 1.088 | 1.539 | 0.158 | 0.224 | 1.443 | 2.041 |
| Effect size, d = | μ_1_ - μ_2_| / σ | 1.148 | 0.812 | 1.051 | 0.741 | 0.907 | 0.641 |
| Test significance level, α | 0.01 | 0.01 | 0.01 | 0.01 | 0.01 | 0.01 |
| Power ( % ) | 80 | 80 | 80 | 80 | 80 | 80 |
| **n per group** | **20** | **38** | **23** | **45** | **31** | **59** |
| Test significance level, α | 0.05 | 0.05 | 0.05 | 0.05 | 0.05 | 0.05 |
| Power ( % ) | 80 | 80 | 80 | 80 | 80 | 80 |
| **n per group** | **13** | **25** | **16** | **30** | **21** | **40** |
| Test significance level, α | 0.01 | 0.01 | 0.01 | 0.01 | 0.01 | 0.01 |
| Power ( % ) | 90 | 90 | 90 | 90 | 90 | 90 |
| **n per group** | **25** | **47** | **29** | **56** | **38** | **75** |
| Test significance level, α | 0.05 | 0.05 | 0.05 | 0.05 | 0.05 | 0.05 |
| Power ( % ) | 90 | 90 | 90 | 90 | 90 | 90 |
| **n per group** | **17** | **33** | **21** | **40** | **27** | **53** |

*Calculated values have been rounded to three decimal places.

**Assume the following: (1) a common standard deviation σ_1_ and variance σ_1_^2^ based on simulated data or (2) a common variance σ_2_^2^ that is twice that of σ_1_^2^. Example: let σ_1_=1.088, then σ_2_ ^2^= 2σ_1_^2^ = 2.367, and σ_2 =_ 1.539.
